# From metabolic alterations to chronic inflammation: mechanisms and immunoregulation of metabolic reprogramming in COPD

**DOI:** 10.3389/fimmu.2025.1698832

**Published:** 2025-12-02

**Authors:** Siyu Zeng, Yanqiu Zhang, Shiran Li, Zhimin Li, Pengfei Li, Jingxian Xie, Jiao Zhang, Liling Xie, Yong Yang

**Affiliations:** 1 Department of Pharmacy, Sichuan Academy of Medical Sciences & Sichuan Provincial People’s Hospital, School of Medicine, University of Electronic Science and Technology of China, Chengdu, China; 2 School of Pharmacy, Southwest Medical University, Luzhou, China; 3 Sichuan Academy of Medical Sciences and Sichuan People’s Hospital, Chengdu, China; 4 Cheng Fei Hospital, Chengdu, China

**Keywords:** Chronic Obstructive Pulmonary Disease, metabolic reprogramming, inflammation, oxidative stress, airway remodeling, immunometabolism

## Abstract

Chronic obstructive pulmonary disease (COPD) is a prevalent chronic respiratory disease characterized by high prevalence, mortality, and disease burden. Current understanding of COPD pathogenesis primarily focuses on airway inflammation, immune dysfunction, oxidative stress, and protease-antiprotease imbalance. Notably, recent studies have increasingly highlighted the role of metabolic reprogramming in COPD. Metabolic reprogramming refers to cellular adaptation through metabolic pathway alterations in response to environmental stress, enabling physiological or pathological state transitions. This review systematically summarizes COPD pathogenesis, with particular focus on metabolic reprogramming features (glucose, lipid, and amino acid metabolism) in immune cells from COPD experimental models. Furthermore, we analyze the interactions between these metabolic alterations and chronic inflammatory responses, providing new insights into COPD pathogenesis.

## 1 Introduction

Chronic Obstructive Pulmonary Disease (COPD) constitutes a prevalent chronic respiratory disorder, characterized by persistent respiratory symptoms and airflow obstruction. It exhibits a higher incidence among the elderly and male populations, with concentrated prevalence in regions with high smoking rates and air pollution. Recent epidemiological data have documented that the prevalence of COPD among adults aged 40 years and older ranges from 9% to 10% (1, 2). According to the latest statistics from the World Health Organization (WHO), COPD ranks among the top five leading causes of global mortality—accounting for 3.5 million deaths in 2021, or approximately 5% of total global deaths—with its prevalence continuing on an upward trajectory. In urban areas of China, COPD ranks fourth in terms of mortality, while its hospitalization rate also exhibits a year-on-year increase (3, 4).

In addition to its direct harms to individual health, COPD imposes a substantial economic burden on families, healthcare systems, and society, driven by high medical expenditures and lost productivity (5). What makes the situation more complex is that COPD is often comorbid with cardiovascular diseases, diabetes mellitus, pneumonia, anxiety/depression, and other conditions. These comorbidities not only increase patients’ risk of hospitalization, prolong the length of hospital stay, and raise the readmission rate, but also further drive up medical costs, significantly increase the difficulty in disease management, and exacerbate the operational pressure on healthcare systems (1, 5, 6).

## 2 Risk factors for COPD

Tobacco smoking stands as the paramount risk factor precipitating COPD. According to research, smoking accounts for over 70% of COPD cases in high-income countries, whereas in low- and middle-income countries, this proportion ranges from 30% to 40% (3). Upon being inhaled into the human body, tobacco particles activate macrophages, neutrophils, lymphocytes, eosinophils, and dendritic cells, leading to pulmonary inflammatory cell infiltration, mucus hypersecretion, airway remodeling, and emphysema, which ultimately results in impairment of lung function (7). Epidemiological evidence further reveals that more than 15% of smokers develop chronic airway obstruction, while approximately 40% to 70% of heavy smokers progress to COPD (8, 9).

**Figure 1:**
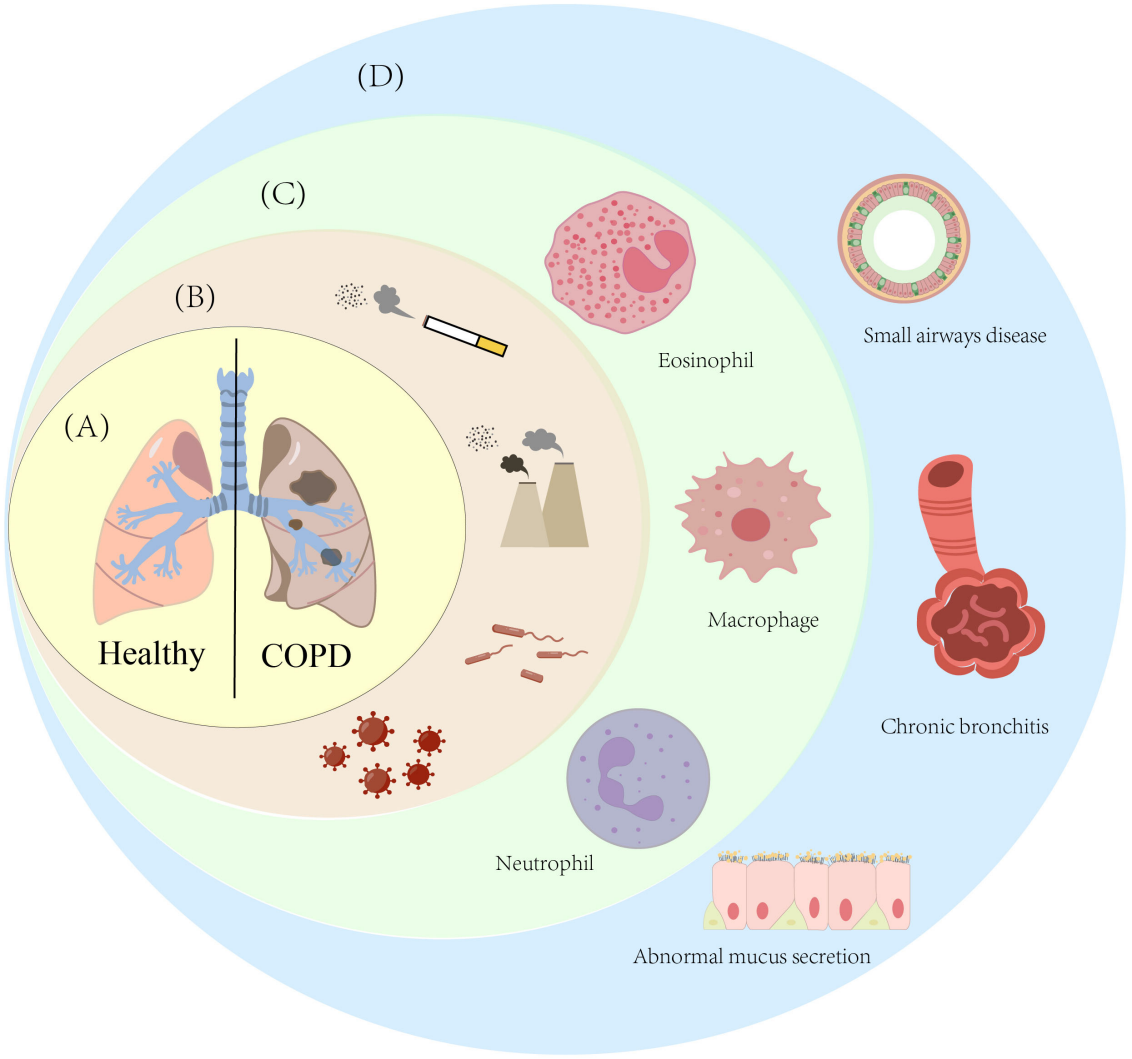
Multidimensional key features of Chronic Obstructive Pulmonary Disease (COPD). **(A)** This diagram shows the comparison of lung tissue between healthy individuals and patients with Chronic Obstructive Pulmonary Disease (COPD); **(B)** This diagram lists the main risk factors for COPD, specifically including smoking, ambient smoke particles, bacterial infections, and viral infections; **(C)** This diagram presents the immune cells associated with COPD; **(D)** This diagram shows the pulmonary pathological changes caused by COPD, mainly including emphysema, airway stenosis, and abnormal mucus secretion by epithelial cells.

Beyond active smoking, other risk factors for COPD also encompass passive smoking, environmental and occupational exposures, infectious factors, an unhealthy diet, and individual factors (such as gender and age differences, as well as genetic factors) (10). Owing to their small particle size and hydrophobicity, persistent environmental particulate matter can easily penetrate terminal bronchioles and alveoli, evade mucociliary clearance, be phagocytosed by alveolar phagocytic cells, and persist in the pulmonary interstitium and epithelial cell membranes for a long time — a process that leads to the formation of carbon-laden macrophages and functional alterations, with these macrophages exhibiting increased volume and enhanced secretion of inflammatory factors (e.g., TNFα, CXCL8) (11). Additionally, particulate matter induces NADPH oxidase-dependent ROS production in macrophages and activates the macrophage-epithelial NF-κB/MAPK pathway, thereby sustaining pro-inflammatory gene transcription and perpetuating chronic inflammation (12). In parallel, external stimuli such as inflammation, infection, hypoxia, or hyperoxia can induce mitochondrial dysfunction, which in turn triggers oxidative stress, inflammasome activation, apoptosis, senescence, and metabolic reprogramming, exacerbating the progression of COPD (13, 14). For instance, hypoxia can drive the transformation of macrophages into the M1 pro-inflammatory phenotype by stabilizing hypoxia-inducible factor-1α (HIF-1α), promote glycolytic reprogramming of activated naive B cells and naive T cells, and simultaneously inhibit the immunosuppressive function of regulatory T cells (Tregs) — thereby triggering and amplifying inflammatory responses. Additionally, HIF-1α upregulates the expression of glucose transporters (e.g., GLUT1) and key glycolytic enzymes (e.g., hexokinase, phosphofructokinase), enhancing cellular glucose uptake and utilization. Cells preferentially rely on glycolysis for energy supply under hypoxic conditions; under oxygen-replete environments, however, preferential glycolysis is only induced and mitochondrial oxidative phosphorylation (OXPHOS) is inhibited when HIF-1α is abnormally activated via PI3K-AKT/oxidative stress and other non-hypoxic pathways. This ultimately exacerbates metabolic disorders and chronic inflammation in COPD, further promoting airway remodeling and progressive decline in lung function (15–17). Epidemiological and experimental evidence indicates that higher dietary fiber intake is associated with a reduced risk of COPD. Unhealthy dietary factors include insufficient dietary fiber intake, a lack of polyunsaturated fatty acids (PUFAs), and other factors. Given that PUFAs possess antioxidant properties against oxidative stress, which may help alleviate airway inflammation, unhealthy diets may indirectly increase the risk of developing COPD by impairing the body’s antioxidant capacity and immune function (18). Meanwhile, gender differences may indirectly influence COPD development by modulating smoking history and occupational exposure patterns; with advancing age, pulmonary function naturally declines, rendering individuals more susceptible to COPD (19). Genetic and epigenetic factors—such as polymorphisms in the HHIP gene locus and α1-antitrypsin deficiency—also play pivotal roles in the onset and progression of COPD (20). Multidimensional key features of Chronic Obstructive Pulmonary Disease (COPD)are shown in **Figure 1**.

## 3 Phenotypes and endotypes of COPD

The clinical phenotype of COPD refers to one or more disease characteristics that reflect differences among COPD patients and correlate with clinical outcomes. COPD is traditionally classified into two types: the chronic bronchitis phenotype and the emphysema phenotype. With the advancement of research, the understanding of COPD etiology has expanded from “exposure to harmful gases/particles” to “multiple etiological types.” New classifications of COPD have emerged, including the pulmonary cachexia phenotype, COPD-bronchiectasis overlap phenotype, rapid progressive phenotype, non-smoking COPD phenotype, comorbidity/systemic phenotype, upper lobe-predominant emphysema phenotype, α1-antitrypsin deficiency phenotype, and early disease-related phenotypes (pre-COPD, early-onset COPD, preserved ratio impaired lung function) (21).

In contrast, an endotype represents the underlying biological or molecular mechanisms that produce these observable traits. Research on COPD endotypes is advancing rapidly, and several specific endotypes have been proposed or identified, including the T2-low endotype (neutrophil-predominant), T2-high endotype (eosinophil-predominant), genetic endotypes (α1-antitrypsin deficiency endotype, telomerase gene mutation endotype), pulmonary vascular endotype, and persistent inflammation/autoimmune endotype (22).

Phenotypes represent the external clinical manifestations of COPD, while endotypes provide mechanistic explanations behind these manifestations, thus linking clinical observations to molecular pathogenesis.

## 4 Key pathological features of COPD

COPD is defined by persistent respiratory symptoms and airflow limitation, with its pathological changes primarily manifesting in three domains: chronic bronchitis, small airway disease, and emphysema.

### 4.1 Chronic bronchitis

Chronic bronchitis is pathologically characterized by hyperplasia of bronchial mucous glands, degeneration and necrosis of airway epithelial cells, squamous metaplasia, and concurrent infiltration of inflammatory cells (e.g., neutrophils, macrophages, and lymphocytes) in the airway mucosa and submucosa. Clinical studies have confirmed that symptomatic chronic bronchitis—marked by chronic cough and sputum production—is associated with increased frequency of acute COPD exacerbations and accelerated decline in pulmonary function (1, 23).

#### 4.1.1 Core cell types and their roles

As the main inflammatory cells infiltrating the airways, neutrophils disrupt the integrity of the airway epithelium by releasing elastase and matrix metalloproteinases (MMP-9), while secreting IL-8 and TNF-α to amplify the inflammatory cascade (24, 25). Activated alveolar macrophages secrete pro-inflammatory cytokines such as IL-6, IL-1β, and TNF-α to mediate chronic airway inflammation; meanwhile, some studies have shown that oxidative stress can inhibit mitochondrial function and enhance glycolytic activity (26, 27). Recent data indicate the involvement of interleukin (IL)-17 and IL-22 in COPD pathophysiology — two cytokines crucial for regulating lung inflammation and infection. During disease initiation and progression, increased IL-17 secretion induces neutrophil recruitment, leading to chronic inflammation, airway obstruction, and emphysema. In the established phase of COPD, an impaired IL-22 response facilitates pathogen-associated infections and disease exacerbations (28). As the core of the airway barrier, damaged airway epithelial cells undergo mucous-secreting cell (goblet cell) hyperplasia, with excessive mucus secretion forming mucus plugs (29).

#### 4.1.2 Key cytokines and regulatory networks

Chemokines (IL-8, CXCL1) are secreted by airway epithelial cells and macrophages to specifically recruit neutrophils to sites of airway inflammation, and their expression is regulated by transcription factors NF-κB and AP-1 (30). The pro-inflammatory cytokine TNF-α initiates the inflammatory cascade, induces the release of other cytokines, and promotes airway epithelial cell apoptosis; IL-6 promotes mucous gland hyperplasia and enhances mucus secretion via the JAK-STAT3 pathway. The reduced expression of anti-inflammatory cytokines (e.g., IL-10) fails to effectively inhibit pro-inflammatory factor production, resulting in sustained inflammatory imbalance (25, 30).

#### 4.1.3 Regulatory roles of transcription factors

NF-κB is persistently activated in airway epithelial cells and macrophages, serving as a core transcription factor mediating chronic inflammation. By binding to the promoters of IL-8 and TNF-α genes, it promotes their expression; meanwhile, it regulates the expression of key glycolytic enzymes (e.g., HK2) to drive metabolic reprogramming (30). Composed of c-Jun and c-Fos, AP-1 is activated by ROS and synergizes with NF-κB to enhance pro-inflammatory factor transcription, while participating in the regulation of airway epithelial cell squamous metaplasia (27).

### 4.2 Emphysema

Emphysema represents another critical pathological hallmark of COPD, defined as “abnormal permanent enlargement of the airspaces distal to the terminal bronchioles, accompanied by destruction of alveolar walls and without significant fibrosis.” Clinically, two primary subtypes predominate: centrilobular emphysema and panlobular emphysema. Centrilobular emphysema, the most common subtype among smokers, predominantly affects the upper lobes of the lungs; panlobular emphysema, by contrast, is often linked to α1-antitrypsin deficiency and primarily involves the lower lobes. Destruction of alveolar walls in emphysematous regions leads to a reduction in the alveolar capillary bed, which diminishes lung elastic recoil—forming the core pathological basis for expiratory dyspnea in affected patients (16).

#### 4.2.1 Core cell types and their roles

Macrophages are key effector cells in emphysema development, degrading elastic fibers of alveolar walls by secreting MMP-9 and MMP-12; meanwhile, macrophages may undergo M1-like metabolic reprogramming. A similar phenomenon has been observed in experimental models succinate accumulation activates HIF-1α, further enhancing glycolysis and pro-inflammatory factor release to accelerate alveolar destruction (27, 31). After infiltrating alveolar walls, neutrophils release elastase and proteinase 3 to degrade elastin and collagen, while producing ROS to damage alveolar epithelial cells; their enhanced glycolytic metabolism increases lactate production, further exacerbating oxidative stress (25). Fibroblasts exhibit decreased proliferative capacity and insufficient collagen synthesis, failing to repair damaged alveolar walls. Their metabolism is dominated by glycolysis, and studies suggest that the PTGES-AKT-HIF-1α pathway may be involved in fibroblast dysfunction, inhibiting their repair function (32).

#### 4.2.2 Key cytokines and metabolic substrate abnormalities

Matrix metalloproteinases (MMP-9, MMP-12) are secreted by macrophages and neutrophils to directly degrade elastic fibers of alveolar walls; reduced expression of tissue inhibitor of metalloproteinases (TIMP) fails to effectively inhibit MMP activity, leading to aggravated tissue destruction (31). Metabolomic studies have shown accumulation of TCA cycle intermediates (e.g., citrate, succinate, α-ketoglutarate) in alveolar tissue, impairing energy metabolism efficiency; decreased sphingomyelin (SM) levels damage mitochondrial membrane fluidity, inhibit OXPHOS, and indirectly promote glycolytic compensation (26, 33).

#### 4.2.3 Regulatory roles of transcription factors

HIF-1α is highly expressed in emphysematous areas, activated by two pathways cigarette smoke-induced ROS and succinate accumulation. It upregulates key glycolytic enzymes (HK2, PKM2) and vascular endothelial growth factor (VEGF), promoting vascular remodeling and alveolar space enlargement, while inhibiting the proliferation and repair of alveolar epithelial cells (27, 34, 35). Nrf2 is a core transcription factor for antioxidant stress. Its reduced activity in COPD fails to effectively clear ROS, resulting in sustained oxidative damage and further exacerbation of alveolar wall destruction (30).

### 4.3 Small airway disease

Small airway disease, however, serves as the primary pathological driver of airflow limitation in COPD. This disorder affects small airways with a diameter < 2 mm, characterized by wall thickening (due to fibrosis and smooth muscle hyperplasia) and luminal narrowing or obstruction (resulting from mucus plug formation and accumulation of inflammatory exudates). Research has demonstrated a significant correlation between the severity of small airway disease and the degree of decline in forced expiratory volume in 1 second (FEV_1_) (36, 37).

#### 4.3.1 Core cell types and their roles

Airway smooth muscle cells (ASMCs) act as the core cells of airway remodeling, exhibiting typical Warburg-like metabolism with abnormal proliferation highly dependent on aerobic glycolysis; studies suggest that HHIP may mediate metabolic reprogramming via PKM2. Decreased HHIP expression relieves inhibition of PKM2, accelerating glycolysis and lactate production to promote cell proliferation (38). Damaged small airway epithelial cells undergo squamous metaplasia and increased mucus secretion; cigarette smoke exposure downregulates their α-enolase, reducing glycolytic efficiency, while activating FAO to produce ROS, exacerbating airway inflammation and fibrosis (39, 40). Lymphocytes B cells produce autoantibodies to participate in chronic inflammation; Th2 cells secrete IL-4 and IL-13 to induce ASMC proliferation and mucus secretion, with glycolysis-dominated metabolism further amplifying airway remodeling signals (7, 41).

#### 4.3.2 Key cytokines and signaling pathways

IL-4 and IL-13 are secreted by Th2 cells and mast cells, activating the STAT6 pathway to promote ASMC proliferation and goblet cell mucus secretion, while upregulating PTGES expression to initiate the PTGES-AKT-HIF-1α pathway. Chemokines (CCL11, CCL24) recruit eosinophils and mast cells to small airways, releasing pro-inflammatory mediators to exacerbate airway narrowing and hyperreactivity. Fibrosis-related factors involve increased synthesis of collagen I and III, secreted by fibroblasts and myofibroblasts, leading to fibrotic thickening of the airway wall; their synthesis is coordinately regulated by TGF-β and HIF-1α.

#### 4.3.3 Regulatory roles of transcription factors

STAT6, activated by IL-4 and IL-13, directly binds to the promoters of ASMC proliferation-related genes (e.g., cyclin D1) and mucus secretion genes (e.g., MUC5AC) to promote their expression, serving as a key transcription factor for small airway remodeling. Decreased activity of forkhead box protein O1 (FoxO1) may fail to inhibit ASMC proliferation and collagen synthesis; its function is regulated by phosphorylation via the AKT pathway, and sustained AKT activation in COPD leads to FoxO1 inactivation.HIF-1α is highly expressed in ASMCs, enhancing glucose uptake by upregulating GLUT1, inducing glycolytic enzyme expression, and inhibiting PDH activity to prevent pyruvate entry into the TCA cycle — providing energy and biosynthetic precursors for ASMC proliferation and myofibroblast differentiation (32, 35, 38).

## 5 Pathophysiological mechanisms of COPD

A complex, multi-dimensional network of mechanisms—among which chronic inflammation and immune response, oxidative stress, protease-antiprotease imbalance, and metabolic dysregulation are widely recognized as core drivers. Intricate crosstalk between these mechanisms collectively propels disease progression.

### 5.1 Chronic inflammation and immune response

Chronic inflammation constitutes a defining pathological feature of COPD, characterized by the infiltration and accumulation of key immune cells (e.g., neutrophils, macrophages, and T cells) in lung tissue and local airways. Concurrently, a spectrum of inflammatory mediators—including cytokines, chemokines, and proteases—is released, collectively establishing a persistent pro-inflammatory microenvironment that sustains disease progression (42). When the body is chronically exposed to harmful particles such as cigarette smoke, the immune response is activated: innate immune cells secrete pro-inflammatory mediators (e.g., tumor necrosis factor-α [TNF-α], interleukin-6 [IL-6], and interleukin-8 [IL-8]), triggering inflammatory reactions and inducing lung tissue damage (43, 44). Adaptive immune cells such as T cells then migrate to lesioned sites, further intensifying the local inflammatory milieu and driving disease progression (44). Throughout this process, the nuclear factor κB (NF-κB) signaling pathway is activated—not only promoting the release of the aforementioned inflammatory factors and inducing local inflammation in the lungs and airways but also potentially contributing to a systemic inflammatory state (45).

In recent years, targeted studies have further unraveled the complex regulatory mechanisms of chronic inflammation in COPD, spanning molecular pathways, cellular effects, animal models, microecological balance, and immune defense.

From the perspective of molecular signaling pathway regulation, He et al. (45) combined clinical samples with animal experiments to demonstrate that the expression levels of microRNA-21 (miR-21), phosphorylated Smad2/3 (p-Smad2/3), and T helper 17 (Th17) cell-related cytokines (IL-17A, IL-6) are significantly upregulated in the lung tissue of COPD patients. When miR-21 was knocked out in mice, cigarette smoke (CS)-induced inflammatory responses were markedly attenuated, and pulmonary function damage was significantly alleviated. This finding clearly indicates that aberrant activation of the miR-21/Smad7/TGF-β signaling pathway promotes the differentiation and maturation of Th17 cells—emerging as a critical molecular mechanism underlying the initiation and persistence of chronic inflammation in COPD (45).

Focusing on the cellular effects of inflammation, a 2017 study by Huang et al. centered on neutrophils. Experiments confirmed that cigarette smoke selectively induces the infiltration and accumulation of neutrophils in lung tissue, while also prompting these cells to release matrix metalloproteinase-9 (MMP-9)—with MMP-9 expression levels increasing significantly with smoke exposure, ultimately triggering emphysematous lesions. Furthermore, the research team successfully recapitulated the pathological process of lung tissue destruction using an elastase-induced disease model, further validating that neutrophil-driven inflammatory responses constitute a core mechanism in the development and progression of emphysema (46).

Shifting to the perspective of airway microecology, a 2024 study by Li et al. was the first to investigate the association between the airway microbiome and COPD inflammation. Analysis of the airway microbiome in COPD patients revealed a significant reduction in α-diversity (microbial richness and evenness), alongside abnormal enrichment of potential pathogenic taxa such as Haemophilus and Pseudomonas. Further research identified synergistic interactions between fungi and bacteria in the airway, which collectively promote the release of pro-inflammatory mediators and exacerbate local inflammatory responses. This study suggests that airway microbiome dysbiosis not only serves as a key driver of chronic inflammation in COPD but may also be closely linked to the frequent occurrence of acute exacerbations (47). Airway microbiota dysbiosis can regulate the synergistic balance of host immunity and metabolism through a dual pathway of “direct metabolic interaction + indirect immune mediation,” driving the pathological progression of COPD. Firstly, the enrichment of pathogenic bacteria (e.g., Haemophilus) leads to the release of pro-inflammatory products such as lipopolysaccharide (LPS), which directly activate host TLR4 signaling pathways. On the one hand, this promotes the release of pro-inflammatory factors including IL-6 and TNF-α (48, 49); on the other hand, it induces enhanced glycolysis and suppressed fatty acid oxidation (FAO) via the phosphorylated AKT/HIF-1α pathway, disrupting glucose and lipid metabolic homeostasis (50, 51). Meanwhile, the reduction of beneficial bacteria (e.g., Bifidobacterium) results in insufficient production of short-chain fatty acids (SCFAs), which not only weakens their anti-inflammatory effects but also loses the inhibitory regulation on glycolysis, further breaking the balance (51, 52). Secondly, microbiota dysbiosis impairs the immune defense function of the airway mucosa, leading to increased bacterial colonization and triggering neutrophil infiltration as well as protease-antiprotease imbalance. Metabolic products such as lactate produced by neutrophils through aerobic glycolysis can reversely amplify macrophage M1 polarization and the release of pro-inflammatory mediators, forming a “immune infiltration - metabolic abnormality - inflammation amplification” feedback loop to indirectly promote metabolic reprogramming (48, 53, 54). Further studies have also found that synergistic interactions exist between fungi and bacteria in the airways, which can jointly promote the release of pro-inflammatory mediators and exacerbate local inflammatory responses. This study suggests that airway microbiota dysbiosis is not only an important driver of chronic inflammation and metabolic disorders in COPD but also closely associated with the frequent occurrence of acute exacerbations of the disease (50, 55).

From the lens of immune defense mechanisms, Richmond et al. (56) focused on the protective role of secretory immunoglobulin A (SIgA). Experiments revealed that mice lacking SIgA exhibited impaired airway mucosal immune defense, leading to abnormal bacterial colonization in the airways and subsequent progressive emphysema. Concurrently, the expression levels of matrix metalloproteinase-12 (MMP-12) and neutrophil elastase were significantly elevated in the lung tissue of these mice—indicating that persistent bacterial colonization in the airways disrupts the “protease-antiprotease” balance in lung tissue, ultimately promoting COPD-like pathological changes. This work provides a novel perspective for understanding the immunoregulatory mechanisms of inflammation in COPD (56).

### 5.2 Oxidative stress

Oxidative stress represents a pivotal mechanism in COPD pathogenesis, defined as an imbalance between the body’s oxidative and antioxidant systems—either due to excessive production of reactive oxygen species (ROS) or impaired antioxidant capacity. Free radicals (e.g., superoxide anions, hydroxyl radicals) present in tobacco smoke serve as direct sources of ROS, exerting direct damaging effects on lung tissue (57). Meanwhile, inflammatory cells such as neutrophils and macrophages—activated by stimuli like smoke particles and dust—produce large quantities of ROS, which in turn activate inflammatory factors such as IL-1β and TNF-α. This forms a positive feedback loop of “ROS—inflammatory factors—ROS” (58). Further compounding this scenario is mitochondrial dysfunction in COPD patients, which also contributes to excessive ROS generation (10, 59).

Oxidative stress directly damages biological macromolecules. ROS can induce oxidative inactivation of antiproteases such as α1-antitrypsin, while simultaneously depleting antioxidant substances like glutathione. This leads to the accumulation of oxidative stress products (e.g., 8-isoprostane), which exacerbates inflammation, damages airway epithelial cells and pulmonary macrophages, and impairs mucociliary clearance function (60, 61). Additionally, ROS activates transcription factors such as NF-κB and AP-1, promoting the release of inflammatory factors; it also regulates mucus-related genes (e.g., Muc5b, Muc5ac), increasing mucus secretion (57, 62).

Oxidative stress further suppresses the antioxidant system. Nuclear factor E2-related factor 2 (Nrf2) regulates the transcription of antioxidant genes; however, CS exposure reduces Nrf2 activity, leading to decreased expression of antioxidant genes and insufficient production of endogenous antioxidants. This impairment of the body’s self-protective mechanisms prevents effective clearance of ROS, ultimately exacerbating lung damage (19, 63).

### 5.3 Protease-antiprotease imbalance

Under physiological conditions, a dynamic balance is maintained between proteases (which degrade tissue components) and antiproteases (which inhibit protease activity) in lung tissue. In COPD, this balance is disrupted—primarily manifested as enhanced protease activity and diminished antiprotease activity (19, 64).

α1-antitrypsin (AAT) represents the most active antiprotease in the lungs, capable of binding to proteases such as neutrophil elastase (NE) at a 1:1 molar ratio to mitigate tissue damage. However, oxidants released by harmful gases (e.g., smoke, dust) induce oxidative inactivation of AAT—with oxidative stress further exacerbating this inactivation (7, 19). Concurrently, smoking disrupts the airway epithelial barrier, prompting activated inflammatory cells to release serine proteases (e.g., neutrophil elastase, proteinase-3) and matrix metalloproteinases (MMPs). These enzymes degrade extracellular matrix proteins (e.g., elastin), leading to lung tissue damage such as collagen degradation (23, 65). Ultimately, antioxidant enzymes fail to effectively inhibit the activity of proteases such as NE and MMPs, resulting in protease-antiprotease imbalance and driving the pathological progression of COPD.

## 6 Metabolic reprogramming in COPD experimental models

### 6.1 Introduction to metabolic reprogramming

The process by which cells alter their metabolic pathways to meet their bioenergetic, biosynthetic, and redox needs in response to adverse environments such as hypoxia and nutrient deficiency is termed metabolic reprogramming (66). In recent years, the rise of metabolomics has provided new ideas and methods for exploring the pathogenesis of COPD (67). Metabolomic studies have revealed abnormalities in multiple metabolic pathways, including amino acid metabolism, lipid metabolism, and glucose metabolism, in COPD patients and experimental models such as cigarette smoke-exposed mice (51, 68), suggesting that metabolic reprogramming is a core component of the pathogenesis of COPD (69). Changes in metabolites are shown in **Table 1**. Underlying this metabolic reprogramming is the abnormal activation of core transcription factors such as HIF-1α and NF-κB, which regulate the expression of genes related to glycolysis, amino acid catabolism, and fatty acid oxidation, respectively (15, 70); epigenetic modifications including methylation and non-coding RNAs further modulate gene activity (66); ROS-mediated inflammatory pathways, microbiota-TLR4 pathways, and disrupted nutrient hormone signaling serve as upstream triggers, with multiple mechanisms synergistically driving the sustained abnormalities of metabolic pathways (51, 68).

**Table 1:** Overview of relevant metabolites and their normal functions in COPD.

Compound name	Main functions	Cell source/Related types	COPD trend (observed in some studies)	Reference
Sphingomyelin (SM)	Maintain cell membrane structural stability and signal transduction	Epithelial cells, serum	Variable	(69, 130)
Glycosphingolipid	Participate in cell recognition and membrane signal regulation	Epithelial cells	Variable	(33, 95, 131)
Polyunsaturated Fatty Acid (PUFA)	Regulate membrane fluidity and exert anti-inflammatory effects	Smooth muscle, epithelial cells	↓	(132, 133)
Lysophosphatidylcholine (LPC)	Inflammatory signaling molecule	Serum	Variable	(134, 135)
Nicotinamide Adenine Dinucleotide Phosphate, Reduced Form (NADPH)	Maintain redox balance and support biosynthesis (main source from PPP)	Various cells (generated via PPP)	↑ (Limited evidence)	(83, 135, 136)
Lactate	Glycolytic product, indicating mitochondrial oxidative impairment or enhanced glycolysis	Skeletal muscle, ASMC, epithelial cells	↑	(137, 138)
Citrate	TCA intermediate, involved in lipid synthesis and metabolic regulation	Mitochondria	Variable	(92, 139)
Succinate	TCA intermediate, acts as a signaling molecule to activate HIF-1α-mediated inflammation	Mitochondria, macrophages	Variable	(140, 141)
α-Ketoglutarate (α-KG)	TCA intermediate, regulates amino acid metabolism and collagen synthesis, participates in glucose-lipid metabolism conversion	Epithelial cells, fibroblasts	Variable/limited	(92, 139, 142)
Glutamate Pyruvate Transaminase 2 (GPT2)	Regulates the conversion of glucose metabolism to lipid synthesis	Epithelial cells	↑	(94, 143)

"↑" indicates increased content of the metabolite in COPD; conversely; "↓" indicates decreased content of the metabolite in COPD.

### 6.2 Glucose metabolic reprogramming

Glucose metabolism encompasses three primary pathways: glycolysis, the pentose phosphate pathway (PPP), and the tricarboxylic acid (TCA) cycle. Glycolysis and the TCA cycle serve as core hubs for energy production, while the PPP primarily supplies the cell with NADPH (to maintain redox balance) and ribose-5-phosphate (for biosynthesis) (71). Glycolysis refers to the 10-step process by which glucose is converted into pyruvate (72). The PPP, by contrast, branches from glucose-6-phosphate, generating NADPH and ribose-5-phosphate (for nucleotide synthesis) (73). In the TCA cycle, pyruvate enters mitochondria and is converted to acetyl-CoA by pyruvate dehydrogenase; acetyl-CoA then enters the TCA cycle, where a series of reactions generate NADH and FADH_2_—substrates utilized in oxidative phosphorylation (OXPHOS) (20).

Abnormal glucose metabolism in COPD patients is not driven by a single factor but rather reflects systemic dysregulation across the three core energy metabolic pathways: glycolysis, the PPP, and the TCA cycle. This dysregulation is driven by environmental factors such as cigarette smoke (CS) and amplified by dysregulated expression of molecules including prostaglandin E synthase (PTGES), Hedgehog-interacting protein (HHIP), and hypoxia-inducible factor-1α (HIF-1α)—forming a vicious cycle of “environmental damage—metabolic reprogramming—pathological progression.”

Aberrant glycolysis in COPD patients serves as a critical metabolic basis for airway remodeling and persistent inflammation. At the systemic level, COPD patients exhibit compensatory enhancement of glycolytic activity even at rest: a study by Kao CC et al. demonstrated that compared to healthy controls, COPD patients display a significantly accelerated rate of whole-body glucose production and a concurrent increase in glucose clearance—indicating enhanced glucose uptake and utilization efficiency. This phenomenon was further corroborated by elevated concentrations of glycolytic metabolites in the vastus lateralis muscle (74). Under exercise stress, non-oxidative metabolism of pyruvate (lactate production) is significantly enhanced, with the magnitude of blood lactate elevation 1.5 to 2 times higher than in healthy individuals. This observation suggests that the TCA cycle cannot meet energy demands under stress, forcing cells to rely primarily on glycolysis for energy production (74). Using Seahorse technology to measure the extracellular acidification rate (ECAR)—a key indicator of glycolytic flux—Cloonan et al. found that human bronchial epithelial cells exposed to CS activate glycogenolysis and upregulate glucose transporter 1 (GLUT1) expression via the phosphatidylinositol 3-kinase (PI3K) signaling pathway within 1.5 hours of rhinovirus infection. This further enhances glycolytic activity, providing essential energy and biosynthetic precursors for viral replication (75). Metabolomic studies have further revealed that serum levels of sphingomyelin (SM) and its hydroxylated form (SM [OH]) are significantly reduced in COPD patients, with SM C24:1 levels exhibiting a negative correlation with glucose clearance. Concurrently, levels of polyunsaturated fatty acid (PUFA)-containing phosphatidylcholine are decreased, while levels of lysophosphatidylcholine are increased. These phospholipid metabolic imbalances impair OXPHOS by disrupting mitochondrial membrane fluidity, indirectly forcing cells to rely on glycolysis for compensatory energy production (76).

At the cell-specific level, distinct lung tissue cells exhibit marked differences in glycolytic phenotypes. Airway smooth muscle cells (ASMCs)—core cells involved in airway remodeling—exhibit a typical Warburg-like metabolic profile, with their abnormal proliferation highly dependent on aerobic glycolysis (38). The differentiation of myofibroblasts also relies on glycolytic reprogramming: stimulation with IL-4, IL-13, or TNF-α upregulates PTGES expression in lung fibroblasts. PTGES then activates the AKT pathway to induce HIF-1α expression, which in turn upregulates the expression of key glycolytic enzymes (hexokinase 2 [HK2], pyruvate kinase M [PKM], and phosphoglycerate mutase 1 [PGAM1]). Treatment with a HIF-1α inhibitor (30 μM) or 2-deoxyglucose (2-DG, 8 mM) completely reverses the PTGES-induced increase in alpha-smooth muscle actin (α-SMA) expression—confirming that glycolysis constitutes an essential step in myofibroblast differentiation (32). Additionally, alveolar macrophages exposed to CS extract (CSE) exhibit suppressed OXPHOS and compensatory increases in glycolysis—a change associated with ROS generation. Treatment with N-acetylcysteine (a ROS scavenger) reverses glycolytic abnormalities and restores macrophage phagocytic function (2). Neutrophils, by contrast, exhibit insufficient glycogen reserves due to decreased expression of glycogen cycle-related enzymes (e.g., glycogen phosphorylase). Pro-inflammatory mediators (e.g., LPS) can induce increased glycolytic flux in neutrophils, with gluconeogenesis compensating for energy demands by generating glycolytic intermediates from non-glucose substrates such as lactate (77). By measuring ECAR and mitochondrial oxygen consumption rate, Malinska et al. observed increased glycolytic flux and OXPHOS damage in human bronchial epithelial cells following smoke exposure (78).

Notably, discrepancies emerge across studies: short-term CS exposure significantly inhibits glycolytic function in type II alveolar cells. CS induces S-glutathionylation of glyceraldehyde-3-phosphate dehydrogenase (GAPDH), leading to its inactivation, reduced ECAR, decreased ATP production, and impaired pyruvate generation (40). A study by SuPing Zhang et al. found that cigarette smoke exposure downregulates alpha-enolase in rat lung tissue, suggesting that this may impair glycolytic efficiency. However, this conclusion lacks direct measurements of glycolytic flux, relying solely on inferences from the downregulation of a single enzyme (79).These different metabolic data also provide new insights for our research: whether abnormal metabolic changes are related to disease progression and the duration of external stimuli.

The core driver of aberrant glycolysis in COPD patients lies in the dysregulation of molecular regulatory networks. Key regulatory axes—including HIF-1α, HHIP-PKM2, PTGES-AKT-HIF-1α, and lactate/LDH-A (LDH5)-TGF-β—interact to collectively promote enhanced glycolysis and diminished oxidative metabolism, sustaining the pathological phenotype of the disease (80–82). Among these, HIF-1α serves as a central transcription factor integrating stress signals and glucose metabolism. Its expression is elevated in COPD lung tissue and can be activated via three pathways: CS-induced ROS activating the PI3K-AKT pathway, macrophage TCA cycle disruption leading to succinate accumulation, and the PTGES-AKT pathway (83, 84). Downstream, HIF-1α upregulates GLUT1 to enhance glucose uptake, induces the expression of glycolytic enzymes, and inhibits pyruvate dehydrogenase (PDH) activity via PDK1—preventing pyruvate from entering the TCA cycle. It also plays a central role in cross-cellular processes such as ASMC proliferation and myofibroblast differentiation (81, 85). The HHIP-PKM2 axis represents a critical link between genetic susceptibility and metabolic abnormalities: mRNA and protein levels of HHIP—a product of the COPD susceptibility gene (4q31 locus)—are reduced by 45% and 30%, respectively, in ASMCs. HHIP deficiency relieves its inhibitory effect on PKM2 catalytic activity and nuclear translocation (80). PKM2 not only accelerates cytoplasmic glycolysis (increasing lactate production by 22%) but also translocates to the nucleus to synergize with HIF-1α in upregulating glycolytic genes. Cigarette smoke further amplifies this effect by inducing PKM2 dimerization via ROS, while overexpression of HHIP reverses glycolytic abnormalities and excessive proliferation in COPD-derived ASMCs. The PTGES-AKT-HIF-1α axis constitutes a core pathway for inflammation-induced glycolysis: pro-inflammatory factors such as IL-4 and IL-13 upregulate PTGES, which catalyzes the conversion of PGH2 to PGE2 to activate the PI3K-AKT pathway downstream of G protein-coupled receptors (32). Activated AKT phosphorylates HIF-1α to inhibit its degradation and promote nuclear localization, ultimately upregulating the expression of glycolytic enzymes—providing energy and collagen precursors for myofibroblast differentiation. Inhibition of PTGES or AKT significantly reduces glycolysis and fibrotic phenotypes.

Abnormalities in the PPP are primarily manifested as oxidative stress-induced compensatory activation. As the rate-limiting enzyme of the pentose phosphate pathway (PPP), glucose-6-phosphate dehydrogenase (G6PD) can promote the production of NADPH through increased activity, and together with PPP, it jointly participates in the regulation of glycolysis and redox balance. Specifically, in 3D bronchial tissues exposed to cigarette smoke, levels of key PPP intermediates (e.g., 6-phosphogluconate, erythrose-4-phosphate) are significantly elevated, while the activity of G6PD, which is the rate-limiting enzyme of the PPP, is enhanced. This directly confirms PPP activation, representing an adaptive cellular response to oxidative stress. However, excessive activation reduces the proportion of glucose entering glycolysis and the TCA cycle, impairing energy production efficiency (20, 86). Short-term CS exposure (4–8 weeks) induces upregulation of PPP-related gene expression in the lung tissue of A/J mice, enhancing PPP activity. This generates reduced nicotinamide adenine dinucleotide phosphate (NADPH) to maintain cellular redox balance and compensate for CS-induced oxidative stress damage—with this change reversible upon cessation of CS exposure (83). These PPP abnormalities ultimately impact cellular function: for example, neutrophils in COPD patients primarily rely on glycolysis for energy, with both the PPP and glycolysis supplying energy and NADPH for the formation of neutrophil extracellular traps (NETs). Cigarette smoke can disrupt neutrophil function by enhancing these two pathways, contributing to the pathological progression of COPD (87, 88).

As the core of energy metabolism, the TCA cycle exhibits abnormalities primarily stemming from insufficient substrate supply and inhibited activity of key enzymes—ultimately leading to reduced energy production efficiency and dysregulation of metabolic intermediates (89). At the systemic level, pyruvate oxidation is usually reduced and lactate levels may be elevated in COPD subjects at rest; during exercise-induced stress, pyruvate non-oxidative metabolism increases disproportionately, and the magnitude of blood lactate elevation is greater than that observed in healthy individuals, which suggests insufficient oxidative reserve of the tricarboxylic acid (TCA) cycle that fails to meet the metabolic demands under stress conditions. Under exercise stress, however, non-oxidative metabolism of pyruvate is significantly enhanced, with blood lactate elevation exceeding that in healthy individuals. This indicates insufficient oxidative reserve of the TCA cycle, preventing it from meeting metabolic demands under stress (74, 90). Cigarette smoke (CS) further exacerbates this abnormality: it induces a 2-fold increase in IRP2 expression, leading to increased mitochondrial iron loading. Iron overload promotes ROS generation and inhibits the activity of aconitase—a key TCA enzyme—resulting in reduced cycle efficiency and citrate accumulation (2). Concurrently, molecular regulatory networks exacerbate substrate insufficiency: elevated LDH5 activity increases lactate production, reducing pyruvate influx into the TCA cycle; HIF-1α further blocks pyruvate entry into the cycle by upregulating PDK1 to inhibit PDH activity (85).

These TCA cycle abnormalities are further characterized by the accumulation of intermediates: in the lung tissue of COPD patients and smoke-exposed mice, TCA intermediates such as citrate, alpha-ketoglutarate, and succinate accumulate—with elevated alpha-ketoglutarate also detected in urine. Mitochondrial dysfunction in COPD patients leads to the accumulation of TCA intermediates (e.g., citrate, alpha-ketoglutarate), while smoke-exposed mice exhibit mitochondrial dysfunction and reduced TCA cycle rate (91–93). Additionally, fumarate and malate accumulate in the lung tissue of COPD patients—further indicators of impaired energy metabolism (93). Glutamic pyruvate transaminase 2 (GPT2) regulates citrate production, influencing the shift from glucose metabolism to lipid synthesis. In airway epithelial cells treated with CS, upregulated GPT2 induces citrate accumulation, promoting phospholipid synthesis and contributing to alveolar structural damage (94).

### 6.3 Lipid metabolic reprogramming

Normal lipid metabolism mainly consists of three core pathways: fatty acid β-oxidation (FAO, a catabolic process), fatty acid synthesis, and cholesterol synthesis (62). In the pathological microenvironment of COPD, lipid metabolism undergoes significant reprogramming, characterized by FAO abnormalities, dysregulated sphingolipid and glycerophospholipid metabolism, and imbalanced regulation of triacylglycerol (TAG) and phospholipid synthesis. Among these, the crosstalk between macrophage phenotypic polarization and FAO dysfunction is a key mechanism driving disease progression (20, 33, 95).

#### 6.3.1 Macrophage phenotypic polarization in lipid metabolism of COPD

Macrophages are core cells regulating inflammation and metabolism in COPD lung tissue, and their phenotypic polarization is tightly coupled with FAO function (96). M1 macrophages are dominated by fatty acid synthesis, with significantly reduced FAO activity. They rely on the glycolytic pathway for energy supply, and even preferentially use glycolysis for energy production under oxygen-replete conditions, while continuously releasing pro-inflammatory cytokines such as IL-1β and TNF-α to amplify local inflammatory responses (97, 98). M2 macrophages are characterized by FAO as the main metabolic feature, with enhanced FAO function tightly coupled to intact mitochondrial oxidative phosphorylation (OXPHOS), efficiently generating energy to support the secretion of anti-inflammatory cytokines such as IL-10 and the function of pulmonary tissue repair.

The proportion of M1 macrophages is significantly increased in COPD lung tissue. Their FAO suppression leads to reduced fatty acid breakdown and lipid accumulation, forming “lipid-laden macrophages.” These macrophages have severely impaired phagocytic function and cannot effectively clear apoptotic cells, foreign bodies, and cigarette smoke particles, further exacerbating the stability of the inflammatory microenvironment; meanwhile, upregulated fatty acid synthesis promotes the release of pro-inflammatory cytokines, continuously amplifying chronic inflammation and accelerating airway remodeling (99, 100).

In COPD, the number of M2 macrophages is reduced and their FAO function is insufficient, their anti-inflammatory and tissue repair capabilities are significantly inhibited. They cannot effectively antagonize the pro-inflammatory effects of M1 macrophages, nor can they repair alveolar structural damage and airway epithelial barrier disruption, ultimately promoting the formation of emphysema and progressive decline in lung function (101).

#### 6.3.2 Abnormal fatty acid oxidation

It should be noted that FAO abnormalities have cell-type specificity — human bronchial epithelial cells exposed to short-term cigarette smoke (CS) or treated with cigarette smoke extract (CSE) show upregulated FAO and increased expression of carnitine palmitoyl transferase 1A (CPT1A), the rate-limiting enzyme of FAO, thereby promoting FAO-mediated ROS production and cell death, and participating in the development of emphysema (33). Differences in FAO between immune cells and upper airway cells are shown in **Table 2**; while FAO suppression is the main feature in macrophages, and these cell-specific differences collectively constitute the complexity of lipid metabolic reprogramming in COPD.

**Table 2:** Comparison table of fatty acid oxidation (FAO) between immune cells and airway epithelial cells.

Cell type	FAO functional features	Downstream pathways/Signaling	Physiological and pathological significance	Reference
Immune cells (macrophages, dendritic cells, Tregs)	Enhanced FAO cooperates with mitochondrial OXPHOS to support NAD^+^/ATP homeostasis and is associated with M2-like macrophage phenotypes; PPAR-γ, PGC-1α, and AMPK are key regulators	Activates PPAR-γ/PGC-1α signaling, promotes lipid droplet mobilization and mitochondrial biogenesis; suppresses NF-κB proinflammatory signaling	Suppresses inflammation, promotes tissue repair and immune tolerance; impaired FAO in COPD macrophages is linked to persistent inflammation	(26, 144–146)
Airway epithelial cells	Under homeostasis, FAO supports barrier integrity and energy supply; under oxidative stress or mitochondrial dysfunction, FAO can produce excessive ROS and amplify inflammation	Excess ROS activates NF-κB and NLRP3 inflammasome pathways; altered lipid metabolism reduces antioxidant capacity	In COPD, dysregulated FAO and mitochondrial dysfunction jointly drive oxidative stress, cytokine release, and epithelial barrier damage	(62, 147–151)

#### 6.3.3 Abnormal sphingolipid and glycerophospholipid metabolism

Plasma sphingomyelin (SM) levels are reduced in COPD patients with emphysema, while glycosphingolipids are associated with acute COPD exacerbations. Glycerophospholipid metabolic abnormalities correlate with airflow obstruction: lysophosphatidylcholine (LPC) levels are elevated, and changes in phosphatidylcholine (PC) levels can distinguish between disease phenotypes (76, 102). Compared to healthy controls, COPD patients exhibit reduced plasma LPC and increased phosphatidylethanolamine (PE) levels (103).

#### 6.3.4 Regulation of triacylglycerol and phospholipid synthesis

GPT2 regulates the synthesis of PC and TAG, participating in CS-induced lipid metabolic reprogramming in airway epithelial cells. Knockout of GPT2 inhibits CSE-induced PC and TAG abnormalities, alleviating lung damage. In smoking-induced lipid metabolic reprogramming, GPT2 regulates the expression of lipid metabolism-related genes (e.g., ACLY, CHPT1, DGAT1) (94). Plasma levels of lipids such as palmitoylethanolamide are abnormal in patients with advanced COPD, and combined detection of these lipids can improve diagnostic accuracy (104).

### 6.4 Amino acid metabolic reprogramming

Metabolic reprogramming in COPD also extends to amino acid metabolism, with abnormalities documented across multiple amino acid pathways (105).

#### 6.4.1 Abnormal metabolism of branched-chain amino acids

Multiple metabolomic studies have indicated that plasma levels of branched-chain amino acids (BCAAs), namely leucine, isoleucine and valine, tend to decrease in some COPD patients, especially those with malnutrition or sarcopenia. This abnormality is associated with nutritional status and muscle metabolic disorders, and may reflect a pathological state of impaired protein catabolism and energy metabolism. However, evidence for a consistent association between BCAA levels and lung function parameters such as FEV_1_ and FEV_1_/FVC remains insufficient, requiring validation in larger-scale and longitudinal cohort studies (106).

In several animal studies of tobacco/smoke exposure, local or systemic amino acid profiles, including certain BCAAs or their metabolites, have been reported to be upregulated. This may reflect specific compensatory metabolic rearrangement or changes in protein metabolism in the early stage of smoking or in the models, but results between animal and human cohorts are not always consistent (93).

#### 6.4.2 Abnormal tryptophan and kynurenine pathway

Tryptophan is the only essential amino acid containing an indole ring, and the kynurenine (KYN) pathway is its major metabolic route. A growing body of evidence has demonstrated that kynurenine pathway activity is generally increased in COPD and its acute exacerbations: the KYN/TRP ratio has been found to be elevated in peripheral blood or respiratory samples in several studies and is associated with inflammatory markers, suggesting a potential role of this pathway in the inflammation-metabolism crosstalk in COPD. *In vitro* and *in vivo* studies have also shown that the rate-limiting enzyme IDO1 can be induced in epithelial cells and immune cells, with its expression regulated by inflammatory signals such as IFN-γ, IL-6 and TNF. Additionally, experimental and review literatures have proposed that HIF-1α and oxidative stress may be involved in the regulation of IDO1, forming a complex interaction network, but complete evidence for each regulatory chain in human COPD airway tissues is still accumulating (107).

In terms of functional mechanisms, kynurenine and its downstream metabolites affect immune cell fate through the aryl hydrocarbon receptor (AhR), including regulation of the Treg/Th17 balance. Moreover, certain downstream metabolites such as quinolinic acid can affect mitochondrial function and oxidative phosphorylation *in vitro* or model systems, thereby promoting metabolic reprogramming. These molecular mechanisms are supported by multiple reviews and experimental studies, but causal evidence in COPD patients needs further refinement (108).

#### 6.4.3 Glutamine and glutamate metabolic imbalance

The importance of glutaminolysis in lung diseases is increasingly recognized. Multiple cellular and animal studies have shown that upregulation of glutaminase (GLS) is associated with metabolic reprogramming and profibrotic phenotypes of lung fibroblasts and macrophages. In pro-inflammatory macrophages (M1), glycolysis is predominant; glutaminolysis can assist in the production of succinate and itaconate, but energy supply still mainly relies on glycolysis. In M2 macrophages, the pathway of glutaminolysis → α-ketoglutarate (α-KG) → complete TCA cycle/oxidative phosphorylation (OXPHOS) is a decisive metabolic feature that supports their anti-inflammatory function. Recent studies have indicated that glutamate-pyruvate transaminase 2 (GPT2) expression in smoking/smoke-exposed airway epithelial cells is associated with metabolic reprogramming. Glutamate levels are elevated in CSE-treated airway epithelial cells. Glutamate-pyruvate transaminase 2 (GPT2, also known as ALT2) regulates glutamate metabolism by catalyzing the conversion of pyruvate and glutamate into alanine and α-ketoglutarate (α-KG). Its effect on citrate production is a downstream indirect effect, and it also participates in the lipid synthesis pathway to regulate smoking-induced metabolic abnormalities in airway epithelial cells, thereby affecting energy homeostasis. Preliminary evidence from cellular, animal, transcriptomic and metabolomic studies for the specific pathological contribution of GPT2 in COPD airway structural cells has emerged in recent years, but more clinical samples and functional validations are needed to establish its strength of action in patients and feasibility as a therapeutic target (109).

#### 6.4.4 Other amino acid abnormalities

In the serum of some COPD patients, arginine and proline levels are decreased—correlating with metabolic phenotypes (76). Elevated levels of glycine, aspartate, and gamma-aminobutyric acid (GABA) have been detected in the lung tissue of COPD patients, while elevated arginine levels are observed in mouse models—with these changes linked to inflammation and oxidative stress Glutamine levels exhibit variability across COPD cohorts but are generally associated with oxidative stress and nitrogen balance regulation (67). Hypoxanthine—related to purine metabolism—represents a potential biomarker for COPD (110).

## 7 Crosstalk between metabolic reprogramming and inflammatory responses

Metabolic reprogramming and inflammatory responses exhibit intimate crosstalk, with oxidative stress serving as a critical hub connecting the two. ROS generated during metabolic reprogramming (e.g., enhanced glycolysis, mitochondrial dysfunction) can amplify inflammatory signals, while inflammation further exacerbates metabolic dysregulation—forming a vicious cycle of “metabolic abnormalities—inflammation—immune dysregulation” (2, 111). Differences between the metabolism of healthy cells and that of COPD cells are shown in **Table 3**.

**Table 3:** Comparison of glycolysis, OXPHOS, fatty acid oxidation, and glutaminolysis metabolic pathways in different cells/samples between healthy and diseased states in COPD.

Cell/Sample	Glycolysis	OXPHOS (Mitochondrial respiration/ATP production)	Fatty acid oxidation (FAO)	Glutaminolysis	Reference
Airway Smooth Muscle Cells (ASMC)	Healthy: Steady-state metabolism, glycolysis controlled; COPD: Glycolysis upregulated, supporting proliferation.	Healthy: Normal function; COPD: Mitochondrial dysfunction, relying on glycolysis.	Healthy: Flexibly utilized; COPD: Lipid metabolic reprogramming, providing substrates for synthesis and hypoxia.	Healthy: Limited utilization; COPD: Pathway enhanced, providing carbon/nitrogen sources.	(152–155)
Airway/Bronchial Epithelial Cells	Healthy: Metabolic balance; COPD: Glycolytic enzymes upregulated, accompanied by EMT/inflammatory signal changes.	Healthy: Normal activity; COPD: Mitochondrial damage, relying on glycolysis.	Healthy: Involved in membrane/signal metabolism; COPD: Altered lipid metabolism (heterogeneous evidence).	Healthy: Repair/synthesis; COPD: Altered amino acid metabolism (study-dependent).	(33, 139, 156–158)
Lung/Airway Macrophages	Healthy: M2 relies on OXPHOS/lipid oxidation, M1 switches to glycolysis; COPD: Metabolic reprogramming promotes inflammation.	Healthy: Normal; COPD: Energy reserve exhaustion, functional defects promote inflammation.	Healthy: Involved in energy metabolism; COPD: Impaired OXPHOS → decreased mitochondrial function, reduced phagocytic clearance capacity.	Healthy: Relies on FAO for energy; COPD: Lipid metabolic reprogramming promotes inflammation.	(77, 159, 160)
Neutrophils	Healthy: Highly dependent on glycolysis for energy and effector functions; COPD: Insufficient energy supply/metabolic dysfunction.	Healthy: Limited function; COPD: Impaired energy reserve, metabolic dysfunction.	Healthy: Limited role; COPD: Inconsistent evidence of changes.	No clear conclusion	(77, 159, 160)
Plasma/Sputum/Systemic Metabolomics	Healthy: Steady-state energy metabolism; COPD: Metabolic reprogramming, abnormal lipid metabolism, energy disorder.	Healthy: Steady state; COPD: Systemic OXPHOS metabolic disorder (heterogeneous markers).	Healthy: Steady state; COPD: Abnormal lipid pathways, FAO/lipid metabolism system affected.	None	(33, 67, 161)

### 7.1 Crosstalk between metabolites and inflammatory signaling pathways

Metabolic reprogramming induces cells such as airway smooth muscle cells and epithelial cells to enhance aerobic glycolysis, producing large quantities of lactate. As a core metabolic product, lactate may promote the release of inflammatory mediators (e.g., IL-6, TNF-α) by activating the TGF-β signaling pathway, while enhancing NF-κB pathway activity to exacerbate local inflammatory infiltration in lung tissue — but this direct activation has not been demonstrated in COPD airway cells. Conversely, while inflammatory mediators can induce metabolic reprogramming, the fundamental driver of persistent inflammation lies in metabolic reprogramming—with metabolites such as lactate sustaining and amplifying inflammatory states to form a pathological loop (112, 113).

Metabolic reprogramming induces lipid metabolic dysregulation, increasing free fatty acid production and activating the TLR4 signaling pathway—directly exacerbating inflammatory responses. Concurrently, lipid metabolic reprogramming induces the synthesis of lipid mediators such as prostaglandin E2, further promoting the production of pro-inflammatory factors like IL-6 and aggravating respiratory inflammation. Additionally, lipid-laden macrophages have been observed in COPD patients, and their core functional abnormalities are mainly manifested by impaired phagocytic function and altered cell survival rate. Notably, the association between lipid accumulation and reactive oxygen species (ROS) is indirect; to date, no clear evidence has been found that it directly induces ROS production, and the amplification effect of related inflammatory damage may need to be mediated through other downstream pathways (114).

Metabolic reprogramming can regulate the expression of metabolism-related genes (e.g., thioredoxin reductase 1, TXNRD1), which exerts redox/antioxidant regulatory functions and participates in COPD-related metabolic disturbance processes by regulating redox balance (115). Concurrently, metabolic reprogramming induces high expression of MMP-9 (supported by increased synthetic precursors from enhanced glycolysis), which activates TGF-β1—contributing to both elastin degradation and fibrosis. This establishes a “metabolically driven link” between inflammation and tissue damage (113).

### 7.2 Metabolic reprogramming and immune cell function

Metabolic reprogramming shifts macrophage metabolism toward aerobic glycolysis, directly promoting M1-type pro-inflammatory polarization. M1 macrophages rely on glycolysis to rapidly generate ATP and pro-inflammatory factors (e.g., IL-1β, TNF-α), while simultaneously producing large quantities of ROS. Disruption of metabolic reprogramming (e.g., glycolysis inhibition) significantly attenuates M1 polarization and alleviates inflammatory responses. Cigarette smoke can exacerbate the pro-inflammatory function of macrophages by inducing metabolic reprogramming (enhanced glycolysis)—forming a cascade of “metabolic remodeling—macrophage activation—inflammation enhancement” (2, 116).

Metabolic reprogramming regulates lipid metabolic pathways, directly influencing the differentiation balance of T cell subsets. By modulating fatty acid oxidation (FAO) and lipid synthesis, metabolic reprogramming can promote the differentiation of either anti-inflammatory Treg cells or pro-inflammatory Th17 cells: a shift toward FAO enhances Treg cell differentiation, alleviating inflammation; conversely, a bias toward lipid synthesis increases Th17 cell differentiation, exacerbating inflammation. This metabolically regulated imbalance in T cell differentiation represents a key contributor to immune dysregulation in COPD (117).

Metabolic reprogramming reduces the expression of glycogen cycle-related enzymes in neutrophils, leading to insufficient glycogen reserves—directly impairing their bactericidal capacity and survival time. While pro-inflammatory mediators can induce neutrophils to generate glycolytic intermediates from non-glucose substrates via gluconeogenesis (a compensatory mechanism of metabolic reprogramming), this compensation fails to reverse functional deficits. Ultimately, this impairs the ability of neutrophils to clear pathogens in COPD patients, perpetuating inflammation (77).

## 8 Discussion and prospects

In recent years, with the rapid advancement of technologies such as metabolomics and single-cell sequencing, research on COPD has shifted beyond traditional focus on three classic mechanisms—chronic inflammation, oxidative stress, and protease-antiprotease imbalance. Increasing attention has been directed toward metabolic reprogramming and its complex crosstalk with inflammation and immune dysregulation. It is increasingly recognized that metabolic abnormalities across multiple pathways (e.g., glucose, lipid, and amino acid metabolism) are not merely coincidental manifestations of disease phenotypes but potential drivers of disease maintenance and progression. This paradigm shift has transformed COPD research from a focus on isolated pathological processes to a systemic, multi-dimensional integrative perspective.

Despite the comprehensive review of COPD-related metabolic changes and the complex interactions between metabolic reprogramming, immunity, and inflammation presented herein, several limitations remain. Many theoretical foundations are derived from animal models or small-scale clinical cohorts; metabolic characteristic differences across species may limit the generalizability of conclusions. Additionally, some mechanistic inferences are based on observational data of metabolite levels or single-gene regulation, lacking causal validation via interventional experiments.

Based on the metabolic reprogramming mechanisms elaborated in this review, multiple potential therapeutic directions can be derived. Regarding metabolic inhibitors: HIF-1α inhibitors (e.g., PX-478) can specifically inhibit the enhancement of glycolysis and reduce the release of inflammatory factors (118, 119); inhibitors of key glycolytic enzymes (e.g., HK2, PKM2) may suppress airway smooth muscle cell proliferation and airway remodeling (120); fatty acid oxidation (FAO) modulators (e.g., PPAR-γ agonists) can correct macrophage metabolic imbalance and promote M2 polarization (121, 122). For dietary interventions: branched-chain amino acid (BCAA) supplementation can improve malnutrition and sarcopenia in COPD patients while regulating the mTOR signaling pathway; increased intake of polyunsaturated fatty acids (e.g., Omega-3) can ameliorate lipid metabolism disorders and alleviate inflammatory responses (123–125). In terms of immunomodulators: neutralizing antibodies or receptor antagonists targeting metabolic products (e.g., lactate, kynurenine) can block the “metabolic abnormality-inflammation” vicious cycle (107, 126, 127); regulation of the airway microbiota (e.g., probiotic supplementation) can improve host metabolic and immune imbalances (128, 129). These strategies provide new insights into the precise treatment of COPD, but their safety and efficacy still need to be verified by large-scale clinical trials.

Looking ahead, the field of COPD metabolism holds vast potential for exploration. Focusing on “dynamic associations between metabolism and disease stages,” longitudinal metabolomic cohort studies can track changes in metabolic profiles of patients from early asymptomatic stages to stable and acute exacerbation phases—identifying core metabolic characteristics at different disease stages and addressing gaps in the current understanding of correlations between disease staging and metabolic abnormalities. On the other hand, integrating metabolomics with genomics to construct molecular subtypes of COPD will better align with the demands of personalized medicine in the modern era. In summary, with advancements in technical methodologies and strengthened interdisciplinary collaboration, substantial breakthroughs in precise subtyping, early diagnosis, and personalized treatment of COPD are anticipated—propelling COPD management into a new paradigm.
